# Supplementary material for: SNX5 suppresses clear cell renal cell carcinoma progression by inducing CD44 internalization and epithelial-to-mesenchymal transition
Source: Mol Ther Oncolytics. 2021 Dec 6;24:87–100. doi: 10.1016/j.omto.2021.12.002 (PMC8717386; doi:10.1016/j.omto.2021.12.002)
Supplement: Document S1. Figures S1–S6 and Tables S1–S3 [file mmc1.pdf]

OMTO, Volume *24*

Supplemental information

SNX5 suppresses clear cell renal cell carcinoma progression by inducing CD44 internalization and epithelial-to-mesenchymal transition

Qingqing Zhou, Jiajun Li, Chao Ge, Jinsi Chen, Wei Tian, and Hua Tian

SUPPLEMENTARY MATERIAL

Supplementary Table S1. The sequences of shRNA Target

Identifier	Forward (5’-3’)
SNX5-1	CCCTCATTGACTATGAGAACT
SNX5-2	CCCGTAGTTCGTCTTTAGTTA
KLF9-1	GCTTTGTATGAGTTGTACTTT
KLF9-3	CTCCCATCTCAAAGCCCATTA

SupplementaryTable S2. The sequences of gene-specific primers used for qRT-PCR vector constructs and ChIP assay

**Gene name**	**Forward (5’-3’)**	**Reverse (5’-3’)**
**Primers for qRT-PCR**		
SNX5	GCACACAAAGACCACACTGC	TGCATCTTCTCTCGAGGACC
CD44	CCATCCCAGACGAAGACAGT	GGTTGTGTTTGCTCCACCTT
Oct4	CTTGCTGCAGAAGTGGGTGGAGGAA	CTGCAGTGTGGGTTTCGGGCA
KLF9	GTGACCACCGAATCTGGGTC	GGCCGTTCACCTGTATGCA
**Primers for vector constructs**		
SNX5(-1297/-30)	GCAAAGCATCTTCCTCCACC	CTCAGCCCGAGTCCAAGATG
**Primers for ChIP**		
ChIP-1	GCCACACAGTCCTAGGTGAC	GCGGTCACAGTAATGGGAC
ChIP-2	GAATGAACCCGTTGCACAGA	AATCCAGACTCCTGCCACAG

Supplementary Table S3. Antibodies used in this study.

Antibody	catalog	Dilution	Company
**For Western blotting**			
SNX5	ab180520	1:500	Abcam
MMP9	Ab38898	1:300	Abcam
E-cadherin	3195T	1:300	CST
N-cadherin	13116T	1:800	CST
Zo-1	8193T	1:500	CST
Claudin-1	13255T	1:500	CST
Snail	3879T	1:500	CST
CD44	Sc-7297	1:300	Santa Cruz
Oct4	CST-2750	1:500	CST
MMP2	10373-2-ap	1:300	Proteintech
MMP7	10374-2-AP	1:300	Proteintech
KLF9	A7196	1:300	Abclonal
β-actin	A3854	1:20000	Sigma
Secondary antibody	HRP conjugated goat anti-rabbit IgG	1:4000	Sigma
Secondary antibody	HRP conjugated goat anti-mouse IgG	1:4000	Sigma
**For Immunohistochemistry**			
SNX5	17918-1-AP	1:50	Proteintech
Ki67	Ab16667	1:50	Abcam
PCNA	Ab29	1:10000	Abcam
CD44	Sc-7297	1:50	Santa Cruz
Secondary antibody	Envision kit (HRP, rabbit/mouse, DAB+)	Ready-to-use	DAKO
**For Immunofluorescence staining**			
E-cadherin	20874-1-AP	1:50	Proteintech
N-cadherin	Ab98952	1:50	Abcam
CD44	Sc-7297	1:30	Santa Cruz
Secondary antibody	Alexa Fluor 488 anti-mouse IgG	1:50	Invitrogen
Secondary antibody	Alexa Fluor 594 anti-rabbit IgG	1:50	Invitrogen
**For flow cytometryanalysis**			
E-cadherin	324107	Ready-to-use	Biolegend
CD44	338805	Ready-to-use	Biolegend

Supplementary Figures

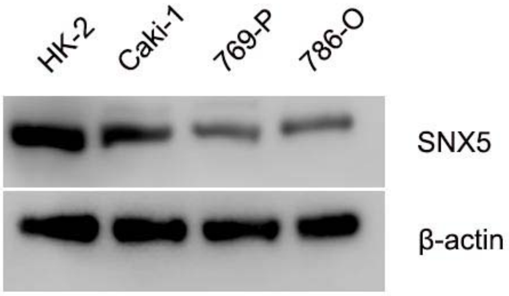

Supplementary Figure S1. The expression of SNX5 was detected by Western blotting in ccRCC cell lines and normal non-malignant renal cell line HK-2.

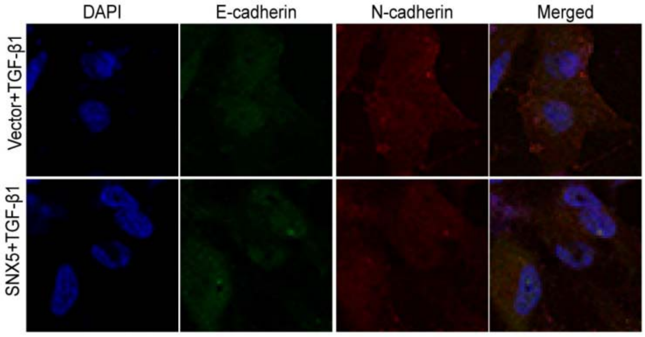

Supplementary Figure S2. SNX5-overexpressing 769-P cells with TGF-β treated were applied to immunofluorescence staining. Antibodies against E-cadherin and N-cadherin were used.

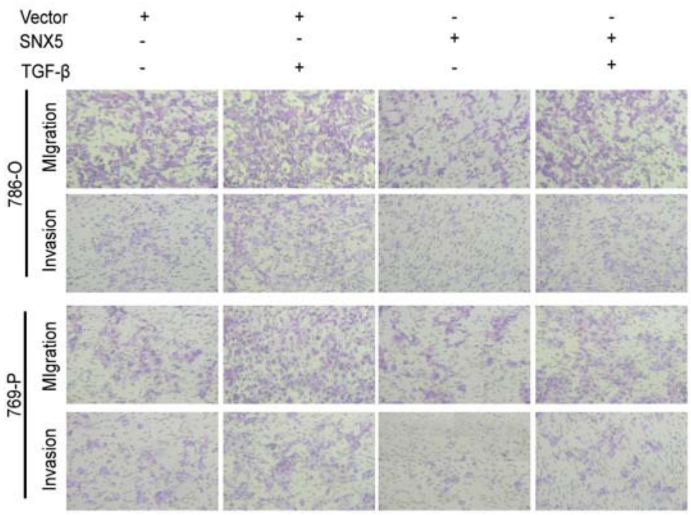

Supplementary Figure S3. SNX5-overexpressed ccRCC cells were treated with TGF-β or control, and cell migration and invasion were evaluated by a transwell assay.

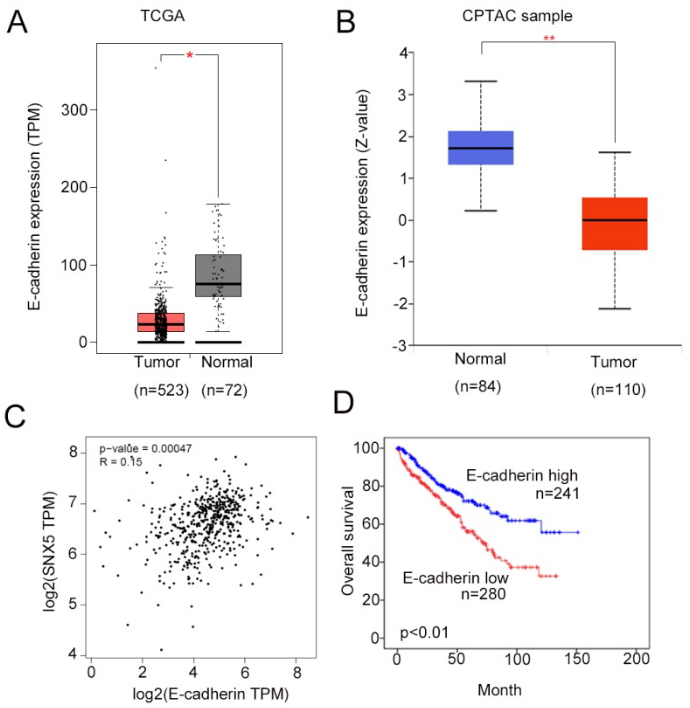

Supplementary Figure S4. **E-cadherin is downregulated and is associated with poor prognosis in ccRCCpatients.**(A) The expression of E-cadherin in ccRCC tissues compared with adjacent normal tissues was analyzed using data sets from TCGA. (B)The expression of E-cadherin in ccRCC tissues compared with adjacent normal tissues was analyzed using data sets from CPTAC. (C) The correlation between SNX5 and E-cadherin in ccRCC tissues using TCGA. (D) Patients with low expression levels of E-cadherin had shorter overall survival than patients with high expression levels as determined using data sets from TCGA.*$p < 0.05$; **$p < 0.01$.

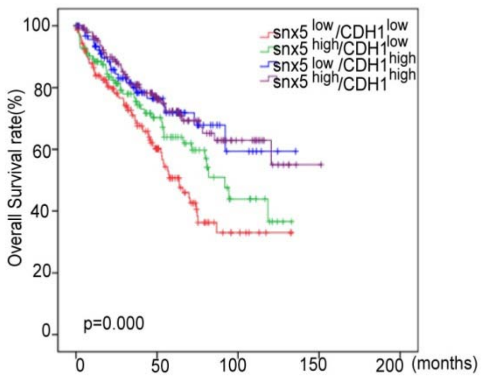

Supplementary Figure S5. Kaplan-Meier analysis of the correlation between the combined expression of SNX5 and E-cadherin with the overall survival of kidney cancer patients according to data sets from TCGA (P<0.01, log-rank test).

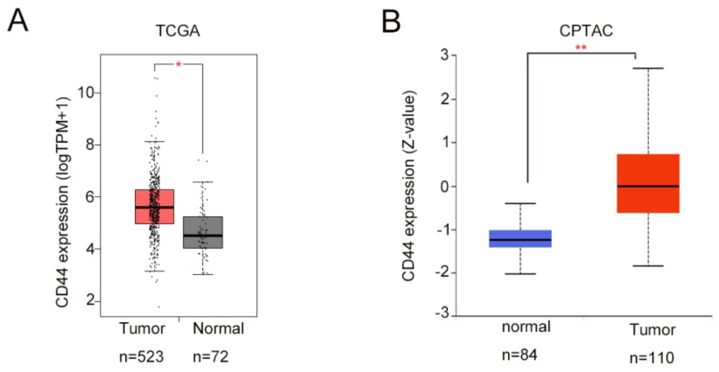

Supplementary Figure S6.(A)The expression of CD44 in ccRCC tissues compared with adjacent normal tissues was analyzed using data sets from TCGA. (B)The expression of CD44 in ccRCC tissues compared with adjacent normal tissues was analyzed using data sets from CPTAC.
