# Supplementary material for: SNX5 suppresses clear cell renal cell carcinoma progression by inducing CD44 internalization and epithelial-to-mesenchymal transition
Source: Mol Ther Oncolytics. 2021 Dec 6;24:87–100. doi: 10.1016/j.omto.2021.12.002 (PMC8717386; doi:10.1016/j.omto.2021.12.002)
Supplement: Document S2. Article plus supplemental information [file mmc2.pdf]

Molecular Therapy
Oncolytics
Original Article

SNX5 suppresses clear cell renal cell carcinoma progression by inducing CD44 internalization and epithelial-to-mesenchymal transition

Qingqing Zhou,[1,2] Jiajun Li,[1,2] Chao Ge,[1] Jinsi Chen,[1] Wei Tian,[1] and Hua Tian[1]

[1]State Key Laboratory of Oncogenes and Related Genes, Shanghai Cancer Institute, Renji Hospital, Shanghai Jiao Tong University School of Medicine, Shanghai, China

Aberrant expression of SNX5 can contribute to tumorigenesis, invasion, and metastasis of several human cancers. However, the clinicopathological and biological significance of SNX5 in clear cell renal cell carcinoma (ccRCC) remain unclear. In this study, we found that SNX5 expression was downregulated and negatively correlated with tumor size, American Joint Committee on Cancer stage, tumor thrombus of inferior vena cava, and poor prognosis in human ccRCC. Ectopic expression of SNX5 inhibited ccRCC cell proliferation and metastasis, whereas knockdown of SNX5 increased these activities both *in vitro* and *in vivo*. Mechanistically, overexpression of SNX5 blocked internalization and intracellular trafficking of CD44 in ccRCC cells. Knockdown of SNX5 was associated with epithelial-to-mesenchymal transition (EMT) in ccRCC cells. Overexpression of SNX5 inhibited TGF-β-induced migration, invasion, and EMT in ccRCC cells. KLF9 directly bound to the SNX5 promoter and increased SNX5 transcription. Moreover, we found that the combination of SNX5 and CD44 or E-cadherin or KLF9 was a more powerful predictor of poor prognosis than either parameter alone. Collectively, our data reveal a mechanism that KLF9-mediated SNX5 expression was associated with poor prognosis via trafficking of CD44 and promoting EMT in ccRCC. SNX5 may be a potential prognostic biomarker and therapeutic target for patients with ccRCC.

INTRODUCTION

Kidney cancer is among the top ten malignant tumors, representing 4.1% of all new cancer cases, and it is estimated that 76,080 people will be diagnosed in 2021 in the United States.[1] Clear cell renal cell carcinoma (ccRCC) is the most common form of kidney cancer, accounting for up to 85% of cases.[2] In recent years, there has been further in-depth understanding of the underlying pathways driving ccRCC biology, resulting in notable improvements in diagnosis and therapeutics.[3] However, owing to recurrence and metastatic diseases, in additional to a lack of effective treatments, the prognosis of ccRCC remains still poor.[4] Therefore, it is urgent to elucidate the molecular mechanism of ccRCC and explore new therapeutic targets.

Sorting Nexin (SNX) is a family that regulates the endocytosis of eukaryotic cells discovered in recent years.[5] This family is relatively conservative in the process of biological evolution, and its structure contains the Phoxhomology (PX) domain, which is tightly combined with phosphoinositol in the membrane structure.[6] As part of a retromer complex, Sorting Nexins (SNXs) play a critical part in transmembrane transport, protein sorting, intracellular signal transduction, and organelle movement.[7] Therefore, SNX family members are closely related to the occurrence and development of tumors. SNX16 expression was significantly upregulated in colorectal cancer (CRC) tissues. Upregulated SNX16 predicted poor survival of CRC patients.[8] SNX10 acts as a tumor suppressor that inhibits colorectal cancer initiation and progression.[9] SNX1 expression is significantly downregulated in colon cancer.[10] Knockdown of SNX1 expression can significantly increase the phosphorylation level of epidermal growth factor receptor (EGFR) and promote colon cancer cell proliferation.[11] SNX6 predicts poor prognosis and contributes to the metastasis of pancreatic cancer cells via activating epithelial-to-mesenchymal transition (EMT).[12] SNX27 promotes breast cancer metastasis, and the elevated expression of SNX27 could be related to the marginally shorter survival of the patients.[13] Our previous research shows that SNX5 predicts poor prognosis and promotes hepatocellular carcinoma progression by modulating the EGFR-ERK1/2 signaling pathway.[14] However, the biological role and molecular mechanism of SNX5 in ccRCC remain unknown. In the present study, we analyzed the expression levels and clinical significance of SNX5 in ccRCC patients and investigated the molecular mechanism underlying its role.

RESULTS

SNX5 is downregulated and correlates with prognosis of ccRCC patients

To explore the role of SNX5 in ccRCC, we first detected the expression of SNX5 using datasets from TCGA (The Cancer Genome Atlas) and

Received 31 August 2021; accepted 4 December 2021;
https://doi.org/10.1016/j.omto.2021.12.002.

[2]These authors contributed equally

Correspondence: Hua Tian, PhD, State Key Laboratory of Oncogenes and Related Genes, Shanghai Cancer Institute, Renji Hospital, Shanghai Jiao Tong University School of Medicine, 25/Ln 2200, Xietu Road, Shanghai 200032, China.
E-mail: htian@shsci.org

This is an open access article under the CC BY license (http://creativecommons.org/licenses/by/4.0/).

GEO (Gene Expression Omnibus). The results showed that the expression of SNX5 was downregulated in ccRCC tissues compared with noncancerous tissues (Figures 1A–1C). To investigate these findings further, we next analyzed SNX5 protein expression using immunohistochemistry and a tissue microarray of patient-derived ccRCC samples (n = 30) and matched normal kidney samples (n = 30). Of the 30 pairs, 24 (80%) had higher SNX5 protein expression in nontumor tissues than in tumor kidney tissues, and 4 (13.3%) had similar expression, whereas only 2 (6.7%) had lower expression (Figure 1D). In addition, expression of SNX5 was also downregulated in ccRCC tumor tissue compared with noncancerous tissues using the Clinical Proteomic Tumor Analysis Consortium (CPTAC) datasets (Figure 1E). Taken together, the results of these analyses show that SNX5 is downregulated in ccRCC.

We next detected the expression of SNX5 in 150 cases of ccRCC using IHC. According to the IHC results, patients were divided into two groups: high and low SNX5 expression groups (Figure 1F). Our results showed that expression of SNX5 was closely associated with tumor size, American Joint Committee on Cancer (AJCC) stage, and tumor thrombus of inferior vena cava (IVC) (Table 1). Kaplan-Meier survival analysis revealed that lower levels of SNX5 were associated with a shorter overall survival (OS) time and higher recurrence rate ($P < 0.01$; Figures 1G and 1I). The same results were also found from the TCGA ccRCC cohort ($P < 0.05$; Figures 1H and 1J). Moreover, univariate and multivariate Cox regression analysis showed that a low level of SNX5 was associated with worse survival of ccRCC patients (Figures 1K and 1L). Therefore,these findings indicate that SNX5 may serve as a valuable prognostic factor for ccRCC patients after surgery.

SNX5 inhibits the proliferation and tumorigenicity of ccRCC cells

The fact that expression of SNX5 is associated with tumor size and tumor thrombus in ccRCC led us to rationalize that SNX5 might be important for ccRCC tumor growth and metastasis. To determine this possibility, 769-P, Caki-1, and 786-O cells were chosen for loss- or gain-of-function studies due to their high or low endogenous SNX5 levels (Figure S1). Results showed the reduced proliferation of ccRCC cells with SNX5 overexpression and enhanced proliferation of ccRCC cells with SNX5 knockdown (Figures 2A–2F).

Next, we examined the effect of SNX5 on the tumorigenicity of ccRCC *in vivo* by using a subcutaneous tumor model in nude mice. As shown in Figure 2G, compared with the control group, 786-O overexpressing SNX5 inhibited tumor growth as determined by tumor volume and tumor weights. In contrast, tumors with shSNX5 exhibited larger volumes and higher weights than tumors with shNC in Caki-1 cells (Figure 2H). Furthermore, expression of Ki-67 and PCNA was significantly reduced in ccRCC cells with SNX5 overexpression and enhanced in ccRCC cells with SNX5 knockdown (Figures 2I and 2J). Taken together, these results provide strong evidence that SNX5 inhibits the tumorigenic ability of ccRCC cells.

SNX5 inhibits invasion and metastasis of ccRCC

To explore whether SNX5 is necessary for ccRCC metastasis, we first analyzed the effects of SNX5 overexpression or knockdown on ccRCC cell migration and invasion. Results showed the reduced migration and invasion of ccRCC cells with SNX5 overexpression and enhanced migration and invasion of ccRCC cells with SNX5 knockdown (Figures 3A and 3B). In addition, we found that overexpression of SNX5 decreased MMP2, MMP7, and MMP9 expression in ccRCC cells, whereas the knockdown of SNX5 increased MMP2, MMP7, andMMP9 expression in ccRCC cells (Figures 3C and 3D).

To determine whether SNX5 expression plays a role in ccRCC metastasis *in vivo*, we injected ccRCC cells expressing Vector or SNX5 into the tail veins of mice to mimic lung metastasis. Histological examination of lung tissues indicated that SNX5 overexpression in tumor-bearing mice had significantly lower numbers of lung metastasis (3/9) than the control group (7/9) (Figure 3E). Taken together, our findings demonstrate that SNX5 plays an important role in the metastasis of ccRCC cells.

SNX5 negatively regulated EMT in ccRCC cells

EMT is a key process by which cancer cells acquire invasive and metastatic properties.[15] Our results demonstrated that SNX5 overexpression in 786-O and 769-P cells resulted in a morphological change of EMT in ccRCC cells, from fiber to round shape, suggesting the inhibition of EMT transition (Figure 4A). In addition, we found that epithelial markers E-cadherin, ZO-1, and Claudin-1 were increased in SNX5 overexpression 786-O and 769-P cells, whereas mesenchymal marker N-cadherin and transcription factor Snail were decreased. On the contrary, knockdown of SNX5 in 769-P and Caki-1 cells decreased the expression of E-cadherin, ZO-1, and Claudin-1 and was accompanied by increased expression of N-cadherin and Snail (Figures 4B and 4C). And, that is the same as the result of western blot on tumor tissues overexpressing and knocking down SNX5 (Figures 4D and 4E). Moreover, we used immunofluorescence to detect the expression levels of E-cadherin and N-cadherin in 769-P cells with stable overexpression of SNX5, and the results were consistent with the findings obtained by western blot (Figure 4F).

We also detected the cell surface expression of E-cadherin in the SNX5-overexpressing and SNX5 knockdown ccRCC cells by flow cytometry. The results showed that SNX5 overexpression increased the expression of E-cadherin compared to the control group in 769-P cells. Conversely, knockdown of SNX5 significantly decreased the expression of E-cadherin in 769-P cells (Figure 4G). Collectively, these results revealed that SNX5 negatively regulates EMT in ccRCC cells.

TGF-β is a secreted cytokine and may function as a tumor promoter by facilitating cancer cells to undergo EMT.[16] Therefore, we investigated whether SNX5 affects TGF-β-induced EMT in ccRCC cells. Our results showed that morphologically, overexpression of SNX5 partially inhibited TGF-β-induced EMT (Figure 4H). Furthermore,

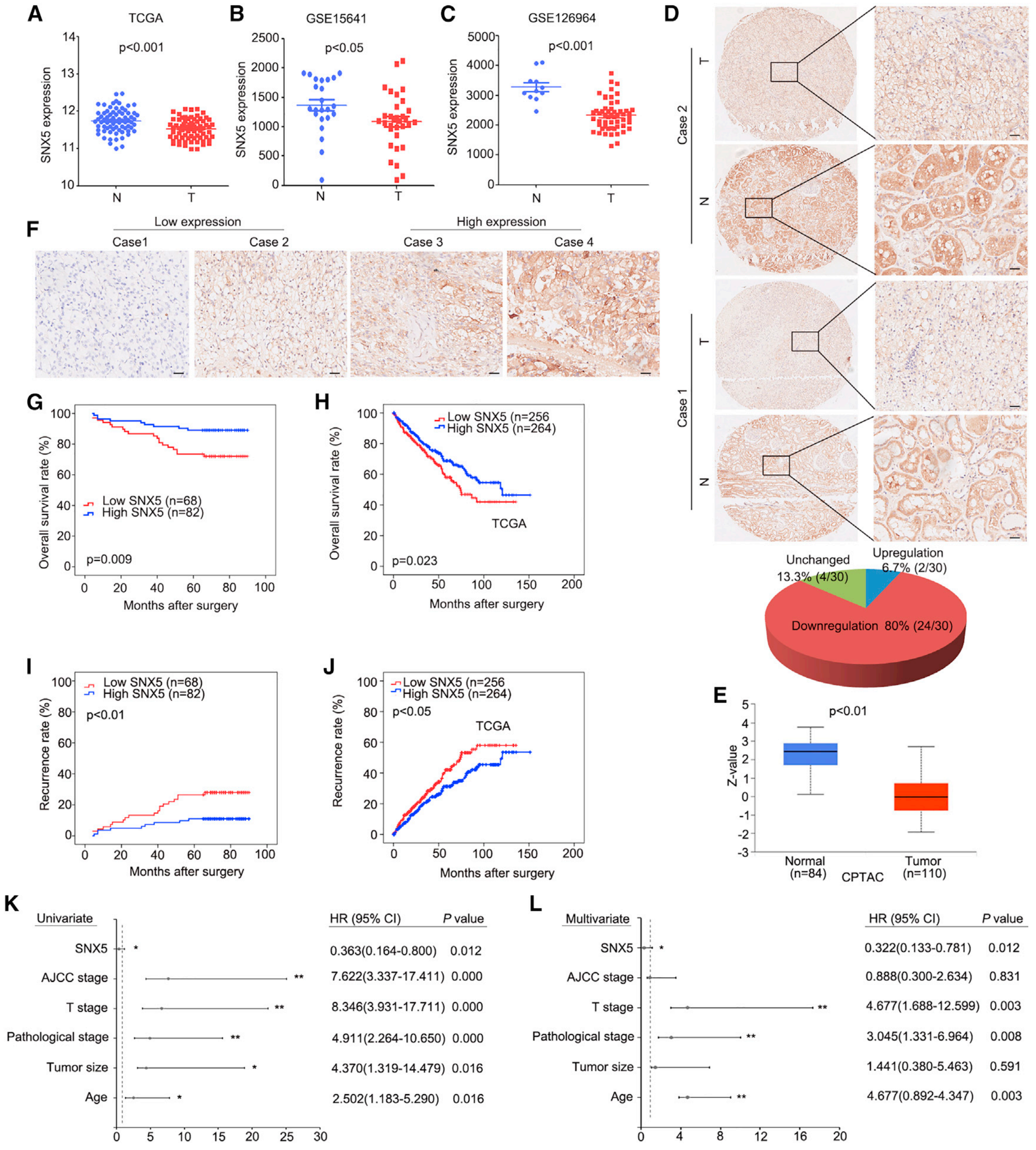

Figure 1. SNX5 is downregulated and associated with poor prognosis in ccRCC patients

(A) The expression of SNX5 in ccRCC tissues compared with adjacent normal tissues was analyzed using datasets from TCGA. (B) The expression of SNX5 in ccRCC tissues compared with adjacent normal tissues was analyzed using datasets from GSE15641. (C)The expression of SNX5 in ccRCC tissues compared with adjacent normal tissues was analyzed using datasets from GSE126964. (D) Representative IHC images showing the expression of SNX5 in ccRCC tissues and adjacent normal tissues. (E) SNX5 protein expression was downregulated in ccRCC tumor tissue using the CPTAC datasets. (F) The expression of SNX5 in ccRCC tissues was cored, and representative IHC images are shown. (G) Kaplan-Meier analysis of overall survival (OS) according to SNX5 expression in 150 ccRCC patients. (H) Patients with low expression levels of SNX5 had shorter overall survival than patients with high expression levels as determined using datasets from TCGA. (I and J) Kaplan-Meier was applied to analyze the association of SNX5 expression and recurrence rate in two HCC cohorts. (K and L) Univariate (K) and multivariate (L) Cox proportional hazards analyses were conducted to evaluate the HR of SNX5 for OS in patients with ccRCC. *p < 0.05; **p < 0.01.

elevated N-cadherin and Snail expression and reduced E-cadherin, ZO-1, and Claudin-1 expression in response to SNX5 overexpression were alleviated by TGF-β treatment (Figure 4I). In addition, we used immunofluorescence to detect the expression levels of E-cadherin and N-cadherin in SNX5 overexpressing 769-P cells treated with TGF-β. The results were consistent with the findings

Table 1. Correlation between SNX5 levels in ccRCC patients and their clinicopathological characteristics

Clinicopathological features	Number	Low expression N (%)	High expression N (%)	p Value
Age				
< 60	95	44 (46.3)	51 (53.7)	0.751
≥ 60	55	24 (43.6)	31 (56.4)	
Gender				
Male	107	51 (47.7)	56 (52.3)	0.366
Female	43	17 (39.5)	26 (60.5)	
Tumor size				
≤3.5 cm	48	16 (33.3)	32 (66.7)	0.043[a]
>3.5 cm	102	52 (51.0)	50 (49.0)	
Pathological stage				
I–II	103	49 (47.6)	54 (52.4)	0.415
III–IV	47	19 (40.4)	28 (59.6)	
T stage				
T1–T2	139	60 (43.2)	79 (56.8)	0.058
T3–T4	11	8 (72.7)	3 (27.3)	
AJCC stage				
I–II	138	59 (42.8)	79 (57.2)	0.031[a]
III–IV	12	9 (75.0)	3 (25.0)	
Tumor thrombus of IVC				
Negative	153	62 (43.4)	81 (56.6)	0.028[a]
Positive	7	6 (85.7)	1 (14.3)	

[a]p< 0.05. IVC, inferior vena cava.

obtained by western blot (Figure S2). Transwell assays showed that overexpression of SNX5 partly reversed the effect of TGF-β on promoting ccRCC cell migration and invasion (Figures 4J and S3). Thus, these data support the view that SNX5 negatively regulates EMT in ccRCC cells.

To further explore potential clinical applications, we next evaluated the expression of E-cadherin in ccRCC using datasets from TCGA and CPTAC datasets. The results showed that expression of E-cadherin was downregulated in ccRCC tumor tissues compared with normal tissues (Figure S4A and S4B). Then, we analyzed the relationship between SNX5 and E-cadherin using datasets from TCGA data. The results showed that there was a positive correlation between the expression of SNX5 and E-cadherin in ccRCC tissue (Figure S4C). Furthermore, Kaplan-Meier survival analysis revealed that lower levels of E-cadherin were associated with a shorter OS time based on TCGA ($P < 0.01$; Figure S4D). Moreover, the patients with low expression of SNX5 and E-cadherin displayed a worse prognosis than the high SNX5 and E-cadherin groups based on TCGA (Figure S5).

SNX5 increases CD44 internalization and affects its recycling

Cancer cells that undergo an EMT acquire cancer stem-cell-like properties and show an increase in CD44 expression.[17] Therefore, we determined the effects of overexpression or knockdown of SNX5 on the marker of potential cancer stem cell (CSC). Our results showed that overexpression of SNX5 inhibited CD44 and Oct4 mRNA and protein expression in 786-O and 769-P cells. Knockdown of SNX5 increased CD44 and Oct4 mRNA and protein expression in 769-P and Caki-1 cells (Figures 5A–5D). And, that is the same as the result of western blot on tumor tissues overexpressing and knocking down SNX5 (Figure 5E).

In addition, our results showed that SNX5 overexpression decreased the cell surface expression of CD44 compared to the control group in 769-P cells, whereas knockdown of SNX5 significantly increased the cell surface expression of CD44 in 769-P cells (Figure 5F). Furthermore, confocal colocalization analysis also revealed that SNX5 overexpression had lower expression of CD44 in 786-O and 769-P cells (Figure 5G). IHC demonstrated that CD44 was downregulated in murine xenografts from SNX5-overexpressing 786-O cells (Figure 6H).

Considering SNX-mediated endosomal transport, we investigated whether the overexpression of SNX5 affects the internalization and recycling of CD44 in ccRCC cells. Our results showed that SNX5 overexpression caused a significant increase in CD44 internalization and affected its recycling to the cell surface (Figures 5I–5K).

Next, we investigated whether SNX5 inhibited ccRCC cell proliferation, migration, and invasion through regulation of CD44. Our results showed that SNX5 overexpression inhibiting cell proliferation, migration, and invasion was partially reversed by the overexpression of CD44 (Figures 6A–6D). Therefore, all results show that SNX5 inhibited ccRCC cell proliferation, migration, and invasion by regulation of CD44.

High expression of CD44 and low expression of SNX5 in ccRCC predicts a poor prognosis

To further explore potential clinical applications, we next evaluated the expression of CD44 in ccRCC using datasets from TCGA and CPTAC datasets. The results showed that expression of CD44 was upregulated in ccRCC tumor tissues compared with normal tissues (Figure S6A and S6B). Furthermore, Kaplan-Meier survival analysis revealed that high levels of CD44 were associated with a shorter OS time based on TCGA ($P = 0.005$, Figure 6E). In addition, the patients with low expression of SNX5 and high expression of CD44 had a worse prognosis than the high SNX5 and low CD44 groups according to TCGA, indicating that the combination of SNX5 and CD44 has better prognostic value than SNX5 or CD44 alone (Figure 6F).

SNX5 is upregulated by KLF9 in ccRCC cells

Our analyses of previously published data from ccRCC samples revealed decreased SNX5 at the mRNA level, leading us to hypothesize that the reduced expression was the result of altered transcriptional regulation. In order to identify potential transcriptional

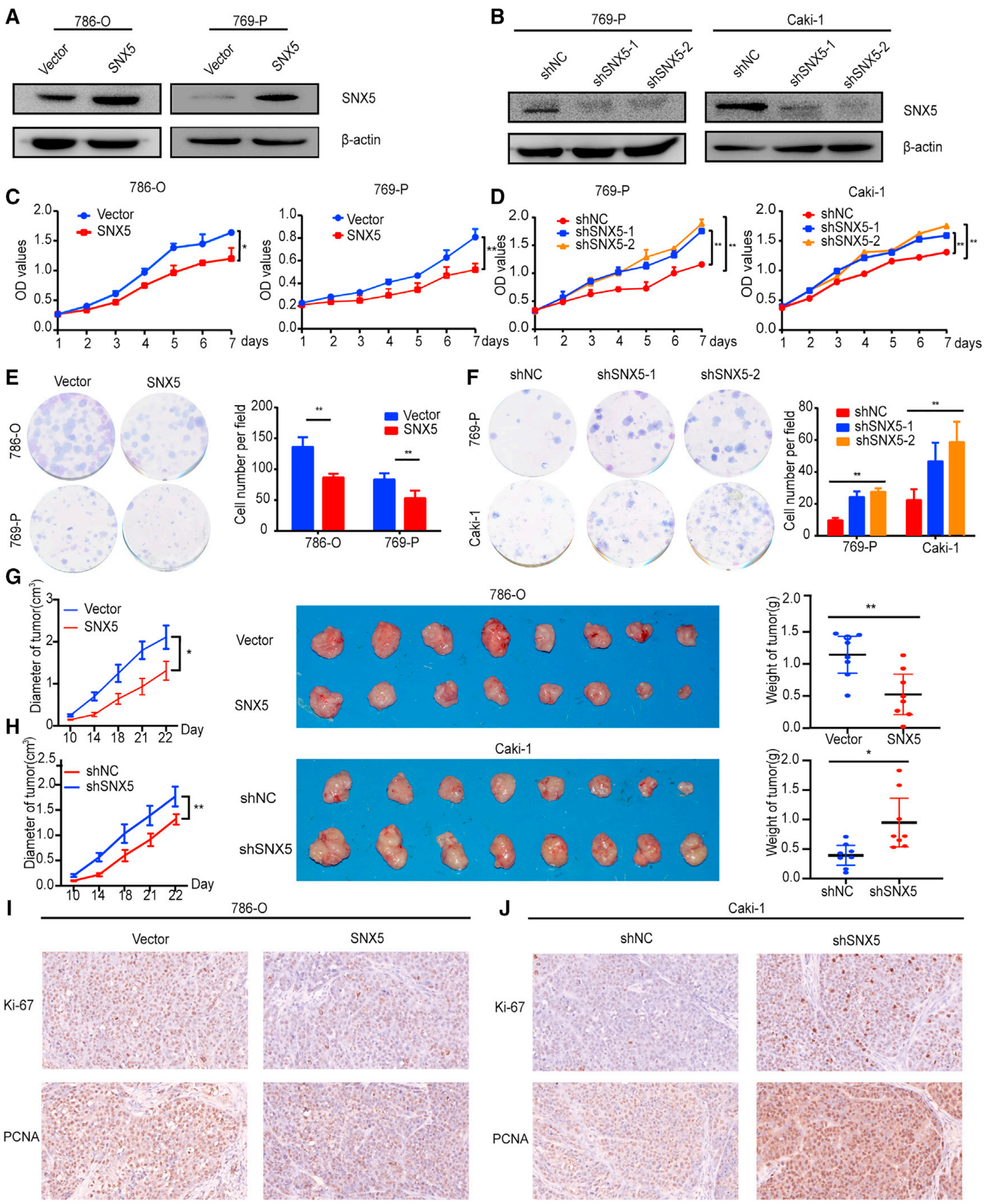

Figure 2. SNX5 inhibits ccRCC cell proliferation

(A) Western blot revealed that SNX5 was efficiently overexpressed in 786-O and 769-P cells. (B) Western blot revealed that SNX5 was efficiently knocked down in 769-P and Caki-1 cells. (C) CCK8 assay demonstrated that the overexpression of SNX5 inhibited CCRCC cells proliferation. (D) CCK8 assay demonstrated that knockdown of SNX5 promoted ccRCC cells proliferation. (E) Colony formation assay showing the proliferation of the SNX5 overexpressed cells. (F) Colony formation assay showing the proliferation of the SNX5 knockdown cells. (G) *In vivo* growth assays showing the difference in tumor diameter and weight between SNX5 overexpressing cells and the control group. (H) *In vivo* growth assays showing the difference in tumor diameter and weight between SNX5 knockdown cells and the control group. (I) IHC assay showing the expression of Ki-67 and PCNA in overexpressing SNX5 ccRCC cells. (J) IHC assay showing the expression of Ki-67 and PCNA in knockdown SNX5 ccRCC cells. *$p < 0.05$; **$p < 0.01$.

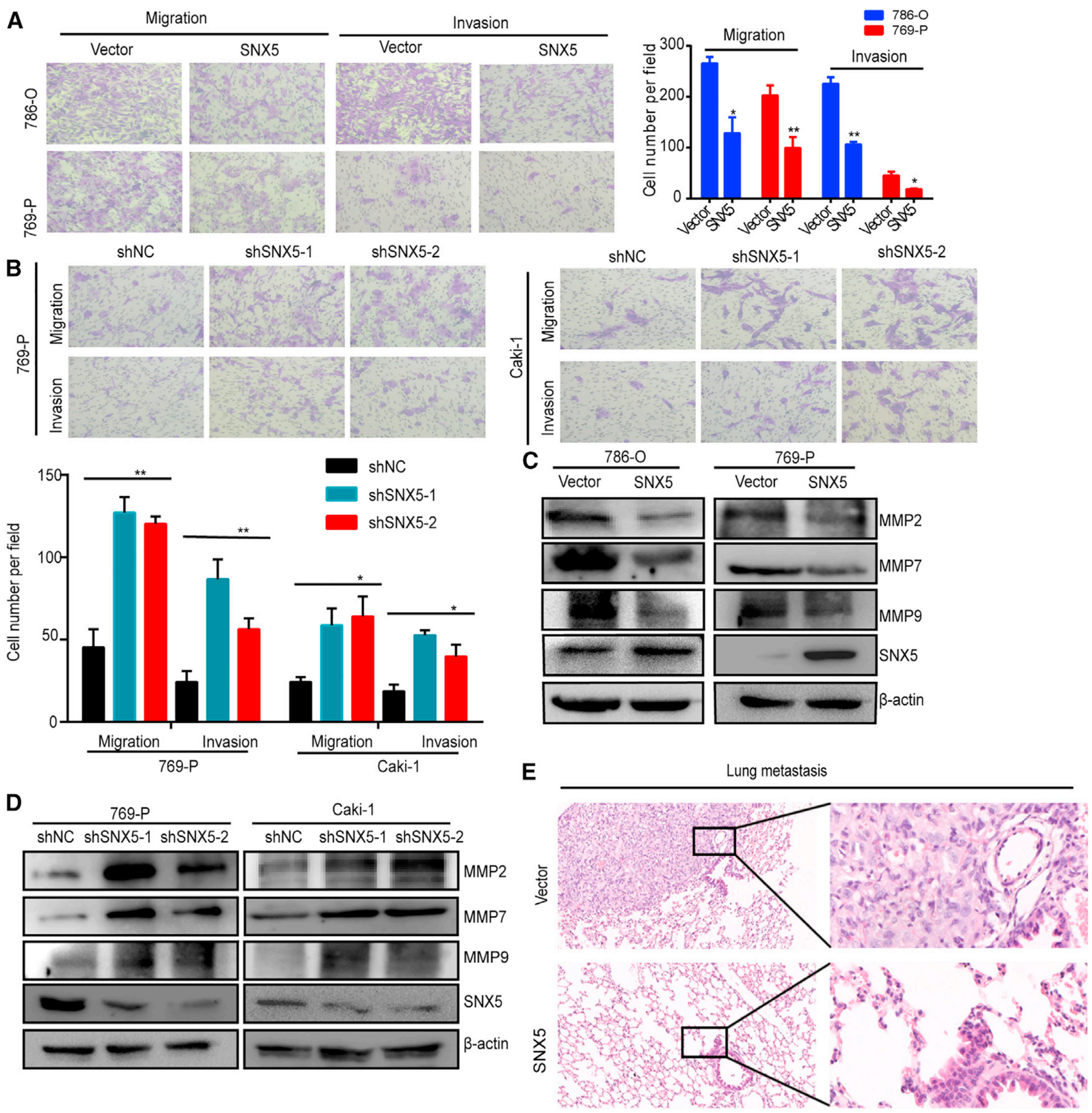

Figure 3. SNX5 inhibits ccRCC cell migration and invasion

(A) Transwell analysis showed that the overexpression of SNX5 inhibited ccRCC cell migration and invasion. (B) Transwell analysis showed that knockdown of SNX5 promoted ccRCC cell migration and invasion. (C) The expression of MMP2, MMP7, and MMP9 was detected by western blot in SNX5-overexpressing cells and (D) SNX5 knockdown cells. (E) H&E staining showing lung metastasis in mice injected via tail vein. *p < 0.05; **p < 0.01.

regulators of SNX5, we performed a reporter assay to identify the regulatory elements that control SNX5 transcription. We analyzed the SNX5 promoter via websites that predicted transcription factor binding sites (JASPAR database). Krüppel-like factor 9 (KLF9) transcription factor binding sites were observed in the promoter of SNX5 (Figures 7A and 7B). KLF9 generally functions as a transcriptional repressor.[18] Luciferase assay showed that knockdown of KLF9 inhibited the activity of SNX5 (Figure 7C). In addition, knockdown of KLF9 inhibited the expression of SNX5 in ccRCC cells (Figures 7D and 7E). The binding of KLF9 to the promoter of SNX5 was further confirmed by ChIP assay (Figure 7F). Furthermore, we found that there a significant positive correlation between the expression of KLF9 and SNX5 in ccRCC tissue (Figure 7G, $r = 0.36$, $P < 0.01$). Therefore, all results suggested that KLF9 binds to the SNX5 promoter and increases its expression in ccRCC cells.

Low expression of SNX5 and KLF9 in ccRCC predicts a poor prognosis

To further explore potential clinical applications of the experimental data, we next assessed the expression of KLF9 in ccRCC based on the

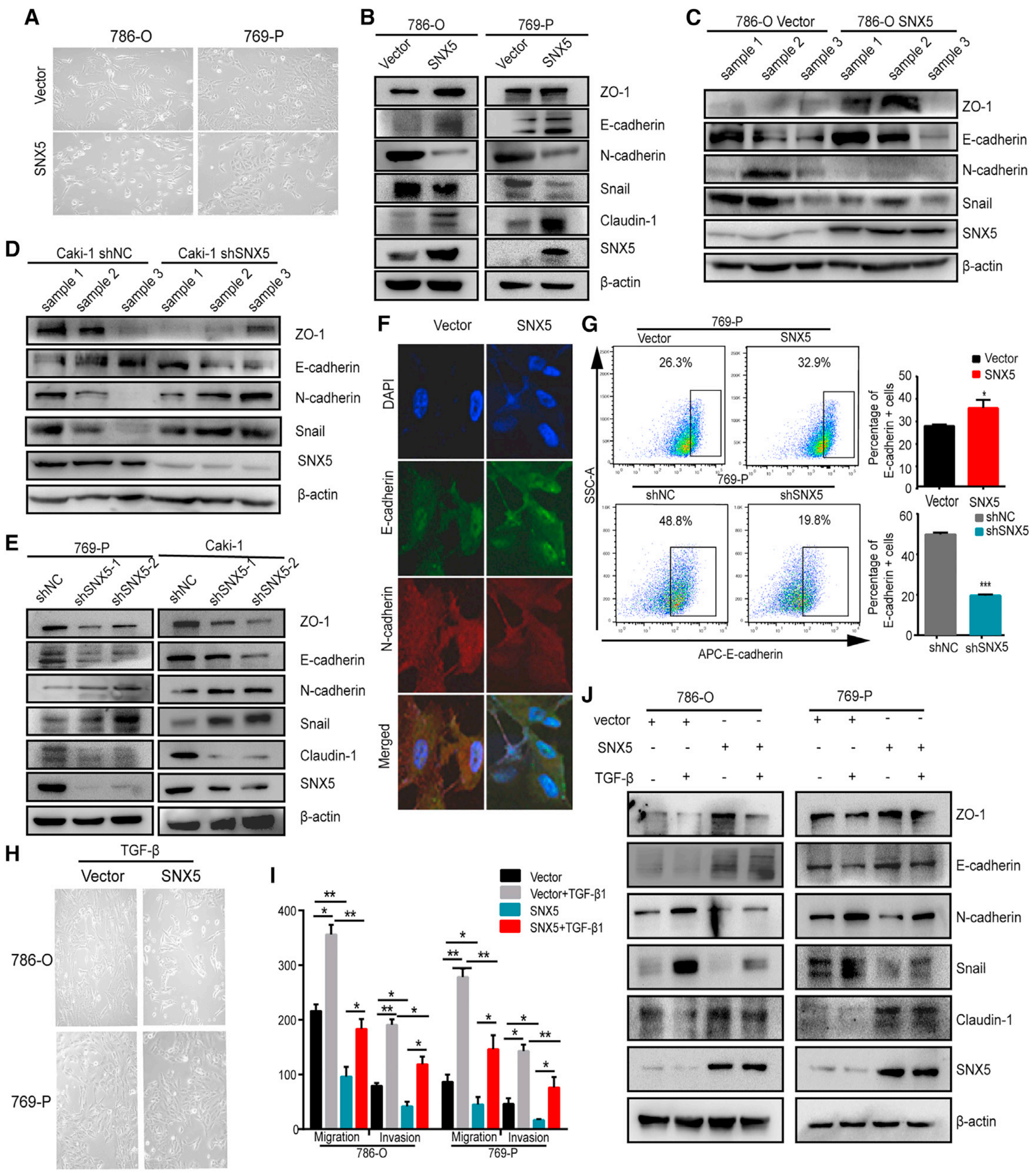

Figure 4. SNX5 suppresses epithelial-to-mesenchymal transition of ccRCC

(A) Representative images of cell morphology in SNX5-overexpressing 786-O and 769-P cells. Western blot analysis of epithelial marker (E-cadherin, ZO-1,Claudin-1) and mesenchymal marker (N-cadherin, Snail) expression in SNX5-overexpressing cells (B) and SNX5 knockdown cells (C). Western blot analysis of epithelial marker (E-cadherin, ZO-1, Claudin-1) and mesenchymal marker (N-cadherin, Snail) expression in SNX5 overexpressing tumor tissue (D) and SNX5 knockdown tumor tissue (E). (F) SNX5-overexpressing 769-P cells were applied to immunofluorescence staining. Antibodies against E-cadherin and N-cadherin were used. (G) E-cadherin on the cell surface in SNX5-overexpressing and SNX5 knockdown 769-P cells was assessed by flow cytometry. (H) Representative images of cell morphology in SNX5-overexpressing 786-O and 769-P cells with TGF-β treated. (I) Western blot analysis of epithelial marker (E-cadherin, ZO-1,Claudin-1) and mesenchymal marker (N-cadherin, Snail) expression in SNX5-overexpressing 786-O and 769-P cells with TGF-β treated. (J) SNX5-overexpressed ccRCC cells were treated with TGF-β or control, and cell migration and invasion were evaluated by a transwell assay.*p < 0.05; **p < 0.01; ***p < 0.001.

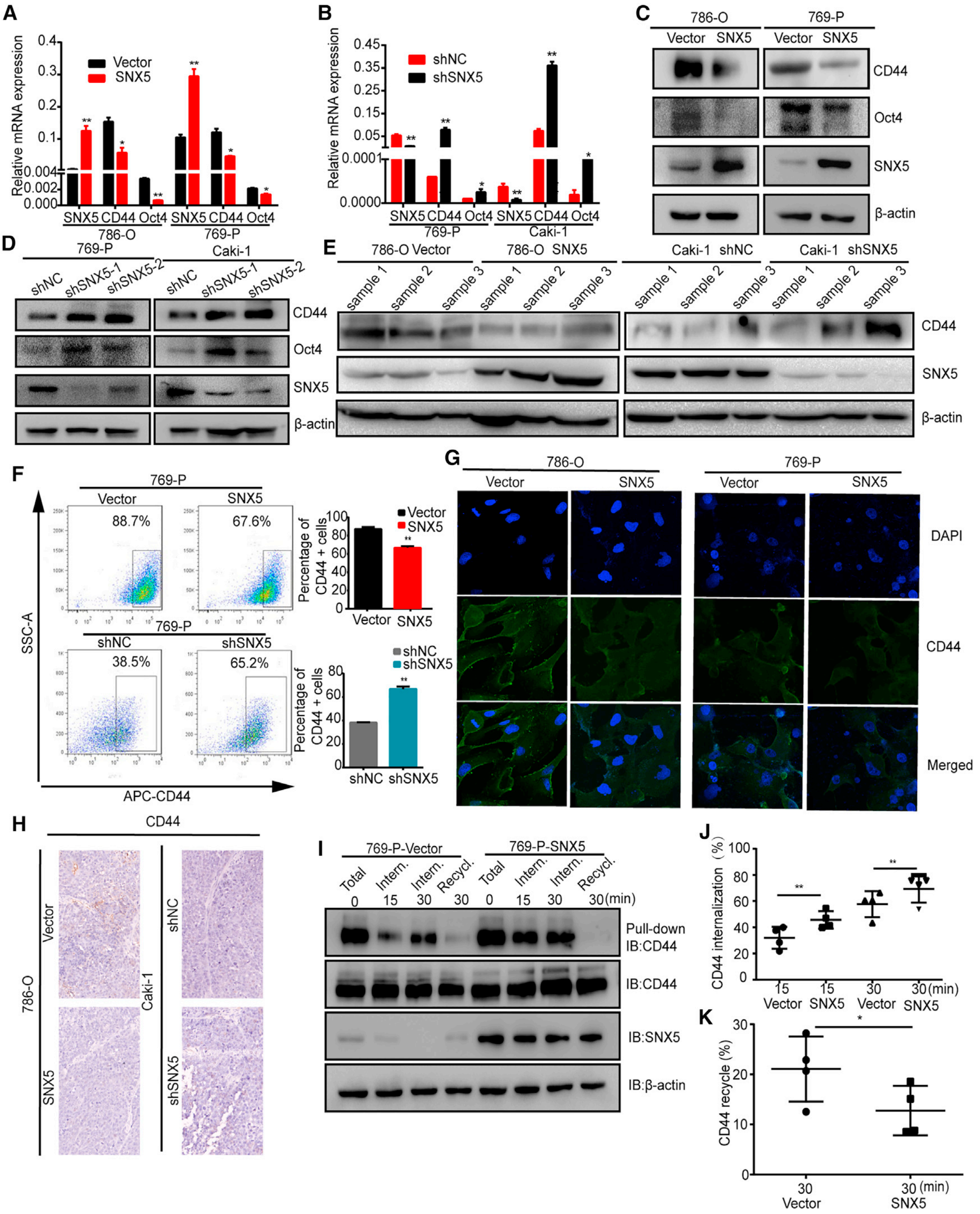

Figure 5. SNX5 inhibits ccRCC cell stemness and CD44 endosomal trafficking

(A) The expression of CD44, Oct4, and SNX5 was detected by qRT-PCR in SNX5-overexpressing 786-O and 769-P cells. (B) The expression of CD44, Oct4, and SNX5 was detected by qRT-PCR in SNX5-knockdown 769-P and Caki-1 cells. Western blot analysis of CD44, Oct4, and SNX5 in SNX5-overexpressing cells (C) and SNX5 knockdown cells (D). (E) Western blot analysis of CD44 and SNX5 in SNX5-overexpressing tumor tissue and SNX5 knockdown tumor tissue. (F) CD44 on the cell surface in SNX5-overexpressing and SNX5 knockdown 769-P cells was assessed by flow cytometry. (G) SNX5-overexpressing 786-O and 769-P cells were applied to immunofluorescence staining. (H) IHC shows the expression of CD44 in SNX5-overexpressing tumor tissue and knockdown SNX5 tumor tissue. (I) Cells were surface-labeled with cleavable biotin at 4°C, left alone to allow internalization or recycling, and examined by western blotting. (J) Quantification of internalized CD44 in SNX5 overexpressing cells from (I). (K) Quantification of recycled CD44 in SNX5 overexpressing cells from (I). $^*p < 0.05$; $^{**}p < 0.01$.

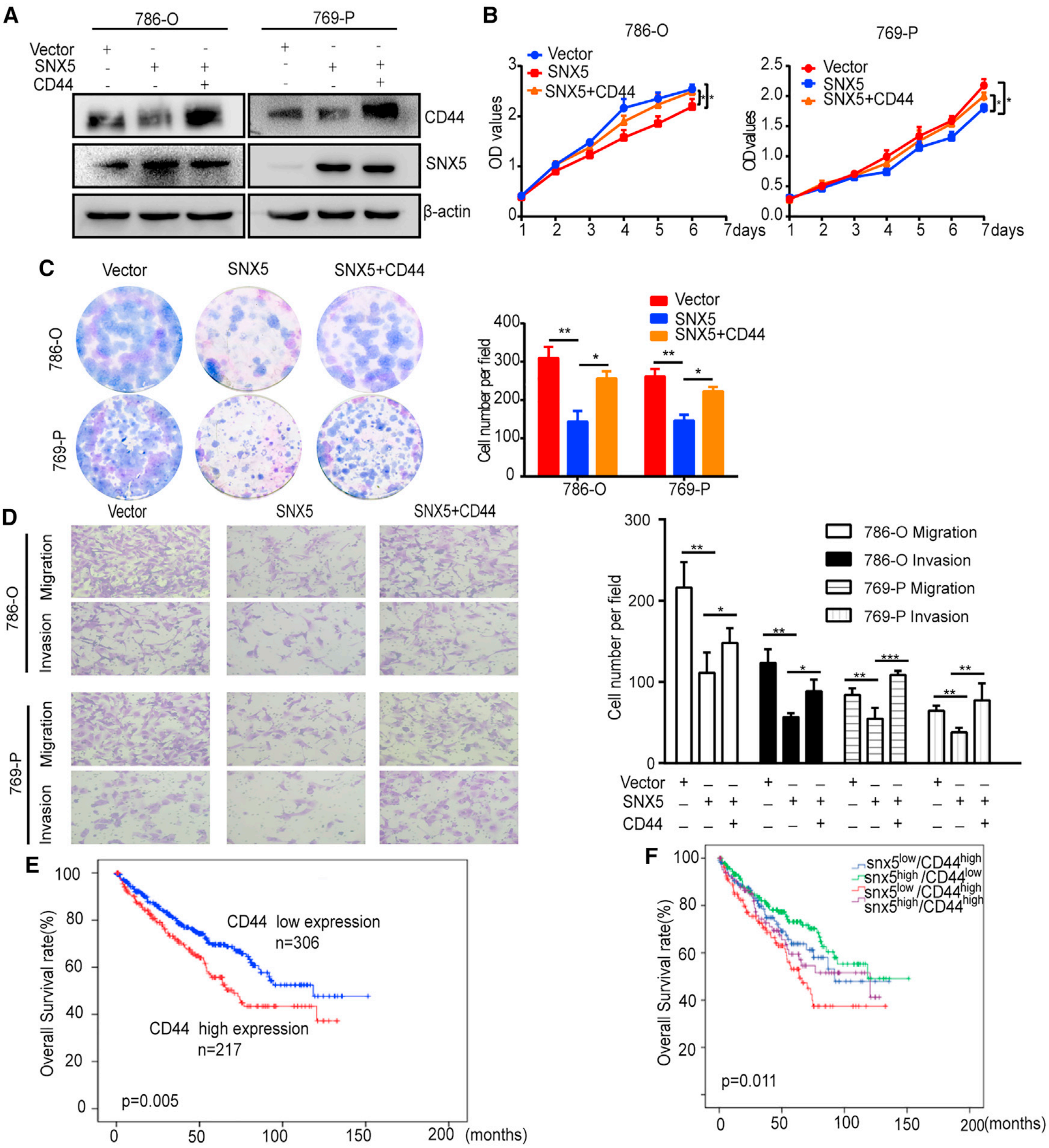

Figure 6. SNX5 suppresses ccRCC cell proliferation, migration, and invasion by CD44 pathway

(A) SNX5-overexpressing 786-O and 769-P cells overexpressed CD44 as indicated, and CD44 and SNX5 expression was detected by western blot. (B) Cell proliferation was assessed by the CCK8 assay in SNX5-overexpressing 786-O and 769-P cells that overexpressed CD44. (C) Cell proliferation was assessed by colony formation assay the in SNX5-overexpressing 786-O and 769-P cells that overexpressed CD44. (D) Cell migration and invasion were assessed by the transwell assay in SNX5-overexpressing 786-O and 769-P cells that overexpressed CD44. (E) Kaplan-Meier analysis showing the overall survival of the high and low expression groups based on CD44 levels according to datasets from TCGA (p = 0.005, log rank test). (F) Kaplan-Meier analysis of the correlation between the combined expression of SNX5 and CD44 with the overall survival of kidney cancer patients according to datasets from TCGA (p = 0.011, log rank test). *p < 0.05; **p < 0.01.

TCGA and GEO. We found that the expression of KLF9 was downregulated in the ccRCC tissues compared with noncancerous tissues (Figures 7H and 7I). Furthermore, the expression of KLF9 was negatively associated with tumor stage, nodal metastasis, and overall survival and disease-free survival (Figures 7J–7L).

Based on the expression of SNX5 and KLF9, ccRCC patients were classified into the following four groups: low SNX5 and low KLF9 group (n = 154), low SNX5 and high KLF9 group (n = 107), high SNX5 and low KLF9 group (n = 118), and the high SNX5 and high KLF9 group (n = 149). The patients with low expression of

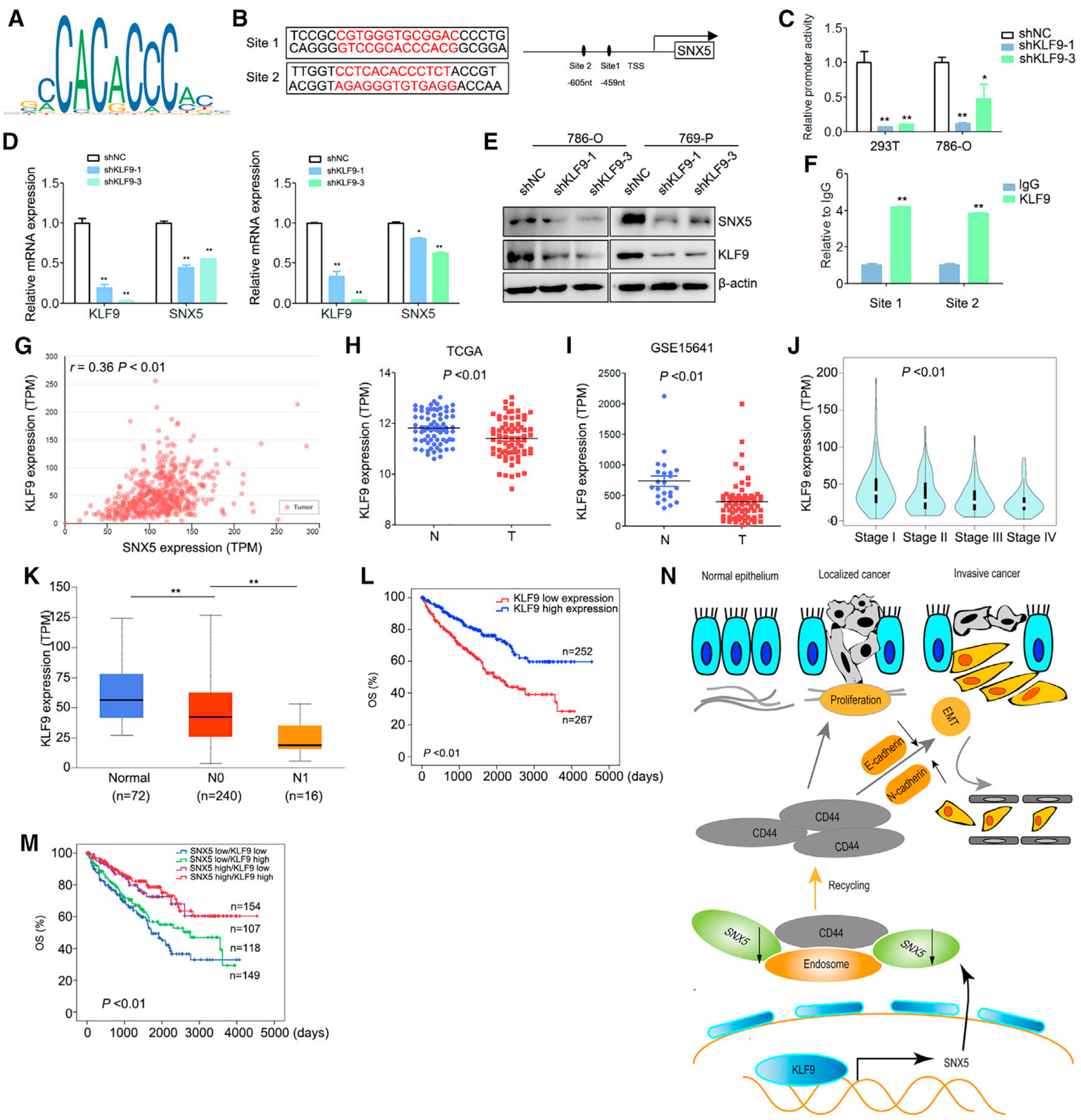

Figure 7. KLF9 binds to the SNX5 promoter and is positively correlated with SNX5 expression in ccRCC tissues and predicts a poor prognosis

(A) KLF9 binding motif. (B) KLF9 DNA-binding sites are present in the human SNX5 promoter region. (C) 786-O and HEK-293T cells were transfected with SNX5 luciferase reporter vectors and KLF9 shRNA. The corresponding luciferase activities were determined by reporter gene assays. (D) The expression of SNX5 mRNA was detected by qRT-PCR in ccRCC cells transfected with KLF9 shRNA or shNC. (E) The expression of SNX5 protein was detected by western blotting in ccRCC cells transfected with KLF9 shRNA or shNC. (F) qRT-PCR for ChIP analysis of KLF9 binding to the SNX5 promoter. (G) The expression of KLF9 was negatively associated with tumor stage using datasets from TCGA. (H) The expression of KLF9 in ccRCC tissues compared with adjacent normal tissues was analyzed using datasets from TCGA. (I) The expression of KLF9 in ccRCC tissues compared with adjacent normal tissues was analyzed using datasets from GSE15641. (J) The correlation between KLF9 expression and tumor stage in ccRCC was analyzed using TCGA datasets. (K) The correlation between KLF9 expression and nodal metastasis status in ccRCC was analyzed using TCGA datasets. (L) OS analysis of patients with ccRCC stratified by the KLF9 expression level using TCGA datasets. (M) Kaplan-Meier analysis of the correlation between the combined expression of SNX5 and KLF9 with the overall survival of kidney cancer patients according to datasets from TCGA ($p < 0.01$, log rank test). (N) Representative model of this study. SNX5 is transcriptionally upregulated by KLF9 in ccRCC. KLF9 deficiency causes a decrease of SNX5 levels, with a consequent increase of CD44 trafficking and recycling, subsequently inducing EMT and promoting cell proliferation, invasion, and metastasis in human ccRCC. *$p < 0.05$; **$p < 0.01$.

SNX5 and KLF9 displayed a worse prognosis than the low SNX5 or KLF9 alone, indicating that the combination of SNX5 and KLF9 has better prognostic value than SNX5 or KLF9 alone (Figure 7M).

DISCUSSION

In the present study, we found that expression of SNX5 is downregulated and associated with a worse prognosis in ccRCC patients. We

found that SNX5 is an inhibitor of proliferation and metastasis in ccRCC cells. Mechanistically, SNX5 facilitates the internalization and inhibits the recycling of CD44 in ccRCC cells, thereby inhibiting EMT of ccRCC cells and exerting a tumor suppressor function.

As a member of the SNX family, SNX5 is involved in endocytosis and internalization of membrane proteins and lipids.[19,20] Endocytosis is an essential component of cell motility. Therefore, endocytosis can influence the transformed cell behavior, including proliferation and metastasis.[21,22] However, the role of SNX5 is different in various tumors. Previous studies showed that loss of SNX5 stabilizes internalized growth factor receptors to promote thyroid cancer progression.[23] However, SNX5 decreases the FBW7-mediated oncoproteins degradation to promote head and neck squamous cell carcinoma progression.[24] Our previous studies showed that expression of SNX5 was upregulated and related with poor prognosis in HCC.[14] Therefore, SNX5 plays different roles in different types of human tumors. In this study, we found that expression of SNX5 was downregulated in ccRCC. The expression of SNX5 was closely associated with tumor size, AJCC stage tumor thrombus of IVC, and prognosis. Moreover, univariate and multivariate Cox regression analysis showed that a low level of SNX5 was associated with worse survival of ccRCC patients. Therefore, these findings indicate that SNX5 may serve as a valuable prognostic factor for ccRCC patients after surgery.

CD44, a non-kinase transmembrane glycoprotein, widely expressed on cell membranes, is implicated in many physiological and pathological processes, which include development, inflammation, immune responses, wound healing, and cancer progression.[25,26] CD44 overexpression or alternative splicing was described for various types of cancers including ccRCC and associated with invasion, metastasis, and resistance to chemotherapeutic drugs.[27–29] CD44 is a known cargo of clathrin-independent endocytosis.[30] Acylation of CD44 is a critical driving force to CD44 association with lipid rafts, which is essential for the rates of hyaluronan endocytosis and CD44 turnover from cell surface.[31] Yu et al. show that receptor-meditated endocytosis by hyaluronic acid for targeting of CD44-overexpressing cancer cells has promising therapeutic prospects.[32] A large endocytic receptor LRP-1 mediated internalization of CD44, which plays an important role in the adhesive properties of tumor cells.[33] Others present that chondrocytes regulate the internalization of CD44, and CD44 turnover is modulated by occupancy with hyaluronan.[34] Here, we demonstrate that overexpression of SNX5 decreased the cell surface expression of CD44 and accelerated internalization of CD44 into the cytoplasm in ccRCC cells. Furthermore, overexpression of CD44 rescued the effects of overexpression of SNX5 on ccRCC cell proliferation and invasion. In addition, the patients with low expression of SNX5 and high expression of CD44 had a worse prognosis than the high SNX5 and low CD44 groups according to TCGA, indicating that the combination of SNX5 and CD44 has better prognostic value than SNX5 or CD44 alone. Therefore, together our data suggest that downregulated SNX5 facilitated the localization of CD44 to the cell surface by recycling, which is an important mechanism to promote ccRCC cell invasion and metastasis.

By regulating multiple important cellular signaling pathways including β-catenin, TGF-β, etc., CD44 is known to promote tumorigenesis and EMT.[35–37] Upregulation of CD44 facilitates EMT-phenotypic change at acquisition of resistance to EGFR kinase inhibitors in lung cancer [38] Our data demonstrate that downregulation of SNX5 decreased the expression of E-cadherin, ZO-1, and Claudin-1 and was accompanied by increased expression of N -cadherin and Snail. Furthermore, knockdown of SNX5 promoted the expression of MMP9 in ccRCC cells. Therefore, the knockdown of SNX5 induces an EMT-like phenotypic transition and promotes metastasis in ccRCC. Furthermore, we found that overexpressed SNX5 inhibited TGF-β-induced EMT in ccRCC cells. TGF-β-induced cell migration and invasion were partially reversed by overexpression of SNX5, suggesting that TGF-β may contribute to these processes downstream of SNX5.

Based on the database analyses and our experiments, we find that KLF9 binds to the SNX5 promoter and increases its expression in ccRCC cells. KLF9 is a zinc-finger-containing transcription factor that belongs to the KLF family and is expressed in various human tissues. KLF proteins have important roles in the regulation of a wide variety of biological processes as well as human diseases, such as cancer.[39] KLF9 acts as a tumor suppressor that has been reported to suppress cell proliferation, migration, and invasion.[40–42] Previous study also shows that KLF9 is downregulated and negatively associated with poor prognosis in human ccRCC.[43] In this study, we find that low expression of KLF9 is one of the reasons for the downregulation of SNX5 in ccRCC. Furthermore, we find that combination of SNX5 and KLF9 has better prognostic value for ccRCC patients. Therefore, downregulated SNX5 by the loss of KLF9 facilitates ccRCC proliferation and metastasis.

In conclusion, this study shows that SNX5 is a tumor suppressor in ccRCC. Mechanistically, knockdown of SNX5 triggers CD44 internalization and recycling, resulting in increased cell surface CD44 expression and promoting cell proliferation and metastasis. Furthermore, expression of SNX5 is regulated by KLF9 in ccRCC cells. The combination of low SNX5, high CD44, and low KLF9 expression may serve as a predictor of poor prognosis in patients with ccRCC (Figure 7N). These findings shed new insights into the pathogenesis of ccRCC and may lead to the development of novel targeted therapies.

MATERIALS AND METHODS

Cell lines and cell culture

786-O, 769-P, and Caki-1were purchased from the Cell Bank of the Chinese Academy of Sciences (Shanghai, China). HK-2 and HEK-293T cell lines were purchased from the American Type Culture Collection (Manassas, VA, USA). 786-O and 769-P were cultured in RPMI 1640 medium, Caki-1 was cultured in McCoy's 5A medium, and ACHN and HEK-293T were cultured in DMEM medium containing 10% fetal bovine serum (FBS), 100 U/ml penicillin and 100 U/ml streptomycin at 37°C with 5% CO_2.

Immunohistochemistry

This study was conducted in accordance with the International Ethical Guidelines for Health-related Research Involving Humans and was approved by the Research Ethics Committee of Renji Hospital. A total of 150 pathologically confirmed ccRCC and 30 adjacent normal kidney tissues were collected by Shanghai National Engineering Research Center from Taizhou Hospital from 2008 to 2015.

Immunohistochemistry (IHC) assays were conducted as reported previously. Briefly, the sections were deparaffinized with xylene and rehydrated before being heated to just below boiling temperature in Tris/EDTA buffer (pH 9.0) for 20 min in a microwave oven for antigen retrieval. The primary antibodies used for the IHC assay were against SNX5. Scores of staining intensity were as follows: 0, negative; 1, weak; 2, moderate; 3, strong. Scores of positively stained cell proportion were as follows: 0, no positive; 1, <10%; 2, 10%–35%; 3, 35%–75%; 4, >75%. The results were scored 0 to 4 by two independent investigators. The individual scores for the staining intensity and percentage of positivity cells were then multiplied to calculate the immunoreactivity score for each sample. Samples having a final staining score of ≤ 4 were considered to exhibit low expression, and those with a score of >4 were considered to exhibit high expression.

Plasmids construction and lentivirus production

The plasmids coding sequences for human SNX5 and CD44 were constructed in our laboratory. The SNX5 lentiviral plasmid was supplied by GeneCopoeia (Guangzhou, China). psPAX2 and pMD2.G plasmids were purchased from Addgene (USA). According to manufacturer's protocol, 293T cells were transfected with a mixture of overexpressing or interfering plasmid, psPAX2 and pMD2.G plasmids for lentivirus packaging using Lipofectamine 2000 (Invitrogen, USA). The viruses were collected after harvesting 48–72 h and added to ccRCC cells with 1×10^6 recombinant lentivirus-transducing units in the presence of 6 μg/mL polybrene (Sigma, USA). The target sequences are listed in Table S1.

Quantitative real-time PCR

Total RNA was retracted with TRIzol reagent (Invitrogen, USA) and was reverse-transcribed with a PrimeScript TM RT Reagent kit (TaKaRa, China). Quantitative real-time PCR (qRT-PCR) was performed with an ABI Prism 7500 System (Applied Biosystems, USA) with SYBR Green Maste (Takara, China). All primer sequences are listed in Table S2.

Western blot

Total proteins extracted from the cells were lysed with RIPA buffer (Thermo Scientific, USA) and separated on 8%–12% SDS-PAGE gels, transferred onto polyvinylidene fluoride membranes (Millipore, USA), incubated with primary antibodies overnight at 4°C, probed with horseradish peroxidase-conjugated secondary antibodies, and visualized using enhanced chemiluminescence reagent (Pierce, USA) via a chemiluminescence analyzer (Bio-Rad, USA). Information on the antibodies is listed in Table S3.

Cell proliferation and colony formation assays

Cell proliferation was measured by the Cell Counting Kit-8 (CCK8) (Bimake, USA) according to the manufacturer's instructions. For colony formation assays, 1,000 cells were plated in each well of a 6-well plate and incubated at 37°C for 2 weeks. Colonies were fixed with 4% phosphate-buffered formalin (pH 7.4) and Giemsa stained for 15 min. Each experiment was performed in triplicate.

Transwell assay

Inoculated 4×10^4 cells in 200 μL of serum-free medium were put in the upper chamber of a transwell (8 μm pore size) or Matrigel-coated transwell upper cavity. The lower chamber contained RPMI 1640 medium with 10% fetal bovine serum as a chemotactic agent. After incubating for 12–24 h at 37°C, we took out the transwell and fixed it with formalin, stained it with crystal violet for 10–15 min, and used a cotton swab to gently wipe off the non-migrated or non-invaded cells from the upper chamber. Five random fields of view were chosen to count under the microscope.

Flow cytometry analysis

To detect the population of E-cadherin$^+$ or the population of CD44$^+$, cells were plated in a 6-well plate and incubated at 37°C overnight, digested with trypsin the next day, washed twice with PBS, and the corresponding antibodies were added and incubated on ice for 45 min, avoiding light. Afterward, the supernatant was discarded, and the cells were washed twice with PBS for flow cytometry analysis. The antibodies used are shown in Table S3.

Immunofluorescence confocal imaging

The cells were inoculated on Lab-Tek laboratory slides one day in advance, fixed with 4% paraformaldehyde at room temperature for 30 min the next day, and washed three times with PBS; then, 0.1% Triton X-100 was permeated for 5 min. They were washed three times with PBS, the primary antibody was added, at 4°C overnight, and washed three times with PBS, incubated in Alexa Fluor 594-conjugated and Alexa Fluor 534-conjugated secondary antibodies and 4′,6-diamidino-2-phenylindole (DAPI) in blocking solution for 30 min at 37°C in a humidified chamber, washed three times, and pictures were taken on a Leica TCS SP8 confocal system (Leica, Microsystems). Information on the antibodies is listed in Table S3.

Internalization and recycling assays

Internalization and recycling assays were conducted as reported previously.[14] We placed the cells at 4°C and washed them twice with cold PBS. We labeled the cell surface with 2 mL of 0.05 mg/mL cleavabe EZ-Link sulfo-NHS-SS-biotin (biotin) at 4°C for 30 min. Unlabeled biotin was washed three times in cold PBS containing 100 mM glycine. We added pre-warmed serum-free RPMI 1640 to the cells and internalized the biotin-labeled surface protein at 37°C at the specified time point, transferring the cells to 4°C to stop internalization. They were washed three times in pre-cooled stripping buffer (reduced 50 mM L-GSH, 75 mM NaCl, 75 mM NaOH, 1% BSA, and 10 mM EDTA, pH 8.0), each time for 10 min to remove surface biotin.

For recycling assays, the biotin-labeled surface protein was internalized at 37°C for 30 min. The surface biotin was removed as described above. After treatment with stripping buffer, the cells were washed once in cold medium. We added pre-warmed, serum-free growth medium and incubated the cells at 37°C for the specified time point. Finally, we incubated the cell lysate with streptavidin-coupled agarose beads at 4°C for 2 h. The agarose bead mixture was washed three times in RIPA buffer, and then the biotinylated protein was detected by western blot analysis.

Luciferase assay

The SNX5 promoter (−1,297 bp/–30 bp relative to the transcription start site) was cloned into the luciferase reporter gene vector pGL3-Basic (Promega). The fidelity of the constructs was confirmed by sequencing. The primer sequences are listed in Table S2.

Cells were cotransfected with the corresponding reporter plasmid and the indicated plasmids in each experiment according to the standard protocol. The pRL-TK reporter construct was used as the internal control. Luciferase activity was detected using a Dual-Luciferase Report Assay (Promega) system in accordance with the manufacturer's instructions.[44]

In vivo growth and metastasis assays

In vivo growth, nude mice were injected with a total of 6×10^6 cells suspended in 200 μL of a mixture of serum-free RPMI 1640/Matrigel (1:1 volume) (BD Biosciences, MA) into the left axillary fossa. For metastasis assay, 2×10^6 cells suspended in 200 μL of serum-free RPMI 1640 were injected into nude mice via tail vein injection. Eight weeks later, the mice were sacrificed, and the tumors and lung tissues were excised and fixed with 4% phosphate-buffered neutral formalin for at least 72 h. Metastatic tissues were analyzed by H&E staining. All of the experiments were approved by the Shanghai Medical Experimental Animal Care Commission.

Statistical analysis

Statistical analyses were performed using SPSS16.0 software. All data are presented as the mean ± SD. Statistical comparisons of the data were performed using two-tailed Student's t test or one-way ANOVA for multiple comparisons. Chi-square tests were used to analyze the data. OS curves were calculated using the Kaplan-Meier method and compared using the log rank test. Univariate and multivariate analyses were performed using the Cox proportional hazard regression model in a stepwise manner. $P < 0.05$ was considered statistically significant.

SUPPLEMENTAL INFORMATION

Supplemental information can be found online at https://doi.org/10.1016/j.omto.2021.12.002.

ACKNOWLEDGMENTS

This work was supported by grants from the National Natural Science Foundation of China (81972581, 82103201), Natural Science Foundation of Shanghai (19ZR1452800), Shanghai Sailing Program (21YF1445400), and Foundation of Shanghai Municipal Health Commission (201940429).

AUTHOR CONTRIBUTIONS

H.T. performed the conception and analyzed data. Q.Q.Z., J.J.L., C.G., J.S.C., and W.T. performed experiments and analyzed data. Q.Q.Z., J.J.L., and H.T. wrote, reviewed, and revised the manuscript.

DECLARATION OF INTERESTS

The authors declare that they have no conflict of interest.

REFERENCES

1. Siegel, R.L., Miller, K.D., Fuchs, H.E., and Jemal, A. (2021). Cancer Statistics, 2021. CA Cancer J. Clin. *71*, 7–33.
2. Klatte, T., Pantuck, A.J., Kleid, M.D., and Belldegrun, A.S. (2007). Understanding the natural biology of kidney cancer: implications for targeted cancer therapy. Rev. Urol. *9*, 47–56.
3. Pantuck, A.J., Zeng, G., Belldegrun, A.S., and Figlin, R.A. (2003). Pathobiology, prognosis, and targeted therapy for renal cell carcinoma: exploiting the hypoxia-induced pathway. Clin. Cancer Res. *9*, 4641–4652.
4. Ljungberg, B. (2013). The role of metastasectomy in renal cell carcinoma in the era of targeted therapy. Curr. Urol. Rep. *14*, 19–25.
5. Mayor, S., and Pagano, R.E. (2007). Pathways of clathrin-independent endocytosis. Nat. Rev. Mol. Cell Biol. *8*, 603–612.
6. Heucken, N., and Ivanov, R. (2018). The retromer, sorting nexins and the plant endomembrane protein trafficking. J. Cell Sci. *131*, 203695.
7. Cullen, P.J., and Korswagen, H.C. (2011). Sorting nexins provide diversity for retromer-dependent trafficking events. Nat. Cell Biol. *14*, 29–37.
8. Shen, Z., Li, Y., Fang, Y., Lin, M., Feng, X., Li, Z., Zhan, Y., Liu, Y., Mou, T., Lan, X., et al. (2020). SNX16 activates c-Myc signaling by inhibiting ubiquitin-mediated proteasomal degradation of eEF1A2 in colorectal cancer development. Mol. Oncol. *14*, 387–406.
9. Zhang, S., Yang, Z., Bao, W., Liu, L., You, Y., Wang, X., Shao, L., Fu, W., Kou, X., Shen, W., et al. (2020). SNX10 (sorting nexin 10) inhibits colorectal cancer initiation and progression by controlling autophagic degradation of SRC. Autophagy *16*, 735–749.
10. Nguyen, L.N., Holdren, M.S., Nguyen, A.P., Furuya, M.H., Bianchini, M., Levy, E., Mordoh, J., Liu, A., Guncay, G.D., Campbell, J.S., and Parks, W.T. (2006). Sorting nexin 1 down-regulation promotes colon tumorigenesis. Clin. Cancer Res. *12*, 6952–6959.
11. Huang, Z., Huang, S., Wang, Q., Liang, L., Ni, S., Wang, L., Sheng, W., He, X., and Du, X. (2011). MicroRNA-95 promotes cell proliferation and targets sorting Nexin 1 in human colorectal carcinoma. Cancer Res. *71*, 2582–2589.
12. Hu, P., Liang, Y., Hu, Q., Wang, H., Cai, Z., He, J., Cai, J., Liu, M., Qin, Y., Yu, X., et al. (2018). SNX6 predicts poor prognosis and contributes to the metastasis of pancreatic cancer cells via activating epithelial-mesenchymal transition. Acta Biochim. Biophys. Sin. (Shanghai). *50*, 1075–1084.
13. Sharma, P., Parveen, S., Shah, L.V., Mukherjee, M., Kalaidzidis, Y., Kozielski, A.J., Rosato, R., Chang, J.C., and Datta, S. (2020). SNX27-retromer assembly recycles MT1-MMP to invadopodia and promotes breast cancer metastasis. J. Cell Biol. *219*, e201812098.
14. Zhou, Q., Huang, T., Jiang, Z., Ge, C., Chen, X., Zhang, L., Zhao, F., Zhu, M., Chen, T., Cui, Y., et al. (2020). Upregulation of SNX5 predicts poor prognosis and promotes hepatocellular carcinoma progression by modulating the EGFR-ERK1/2 signaling pathway. Oncogene *39*, 2140–2155.
15. Thiery, J.P., Acloque, H., Huang, R.Y., and Nieto, M.A. (2009). Epithelial-mesenchymal transitions in development and disease. Cell *139*, 871–890.
16. Hao, Y., Baker, D., and Ten Dijke, P. (2019). TGF-β-Mediated epithelial-mesenchymal transition and cancer metastasis. Int. J. Mol. Sci. *20*, 2767.

17. Mani, S.A., Guo, W., Liao, M.J., Eaton, E.N., Ayyanan, A., Zhou, A.Y., Brooks, M., Reinhard, F., Zhang, C.C., Shipitsin, M., et al. (2008). The epithelial-mesenchymal transition generates cells with properties of stem cells. Cell *133*, 704–715.

18. Knoedler, J.R., Subramani, A., and Denver, R.J. (2017). The Krüppel-like factor 9 cistrome in mouse hippocampal neurons reveals predominant transcriptional repression via proximal promoter binding. BMC Genomics. *18*, 299.

19. Simonetti, B., Paul, B., Chaudhari, K., Weeratunga, S., Steinberg, F., Gorla, M., Heesom, K.J., Bashaw, G.J., Collins, B.M., and Cullen, P.J. (2019). Molecular identification of a BAR domain-containing coat complex for endosomal recycling of transmembrane proteins. Nat. Cell Biol. *21*, 1219–1233.

20. Merino-Trigo, A., Kerr, M.C., Houghton, F., Lindberg, A., Mitchell, C., Teasdale, R.D., and Gleeson, P.A. (2004). Sorting nexin 5 is localized to a subdomain of the early endosomes and is recruited to the plasma membrane following EGF stimulation. J. Cell Sci. *117*, 6413–6424.

21. Poudel, K.R., Roh-Johnson, M., Su, A., Ho, T., Mathsyaraja, H., Anderson, S., Grady, W.M., Moens, C.B., Conacci-Sorrell, M., Eisenman, R.N., and Bai, J. (2018). Competition between TIAM1 and membranes balances endophilin A3 activity in cancer metastasis. Dev. Cell. *45*, 738–752.e6.

22. Chung, B.M., Tom, E., Zutshi, N., Bielecki, T.A., Band, V., and Band, H. (2014). Nexus of signaling and endocytosis in oncogenesis driven by non-small cell lung cancer-associated epidermal growth factor receptor mutants. World J. Clin. Oncol. *5*, 806–823.

23. Jitsukawa, S., Kamekura, R., Kawata, K., Ito, F., Sato, A., Matsumiya, H., Nagaya, T., Yamashita, K., Kubo, T., Kikuchi, T., et al. (2017). Loss of sorting nexin 5 stabilizes internalized growth factor receptors to promote thyroid cancer progression. J. Pathol. *243*, 342–353.

24. Cai, J., Sun, M., Hu, B., Windle, B., Ge, X., Li, G., and Sun, Y. (2019). Sorting nexin 5 controls head and neck squamous cell carcinoma progression by modulating FBW7. J. Cancer *10*, 2942–2952.

25. Orian-Rousseau, V., and Ponta, H. (2015). Perspectives of CD44 targeting therapies. Arch. Toxicol. *89*, 3–14.

26. Pandey, S., Mahtab, A., Rai, N., Rawat, P., Ahmad, F.J., and Talegaonkar, S. (2017). Emerging role of CD44 receptor as a potential target in disease diagnosis: a patent review. Recent Pat Inflamm. Allergy Drug Discov. *11*, 77–91.

27. Li, X., Ma, X., Chen, L., Gu, L., Zhang, Y., Zhang, F., Ouyang, Y., Gao, Y., Huang, Q., and Zhang, X. (2015). Prognostic value of CD44 expression in renal cell carcinoma: a systematic review and meta-analysis. Sci. Rep. *5*, 13157.

28. Wang, A., Chen, M., Wang, H., Huang, J., Bao, Y., Gan, X., Liu, B., Lu, X., and Wang, L. (2019). Cell adhesion-related molecules play a key role in renal cancer progression by multinetwork analysis. Biomed. Res. Int. *2019*, 2325765.

29. Yin, T., Wang, G., He, S., Liu, Q., Sun, J., and Wang, Y. (2016). Human cancer cells with stem cell-like phenotype exhibit enhanced sensitivity to the cytotoxicity of IL-2 and IL-15 activated natural killer cells. Cell Immunol. *300*, 41–45.

30. Eyster, C.A., Higginson, J.D., Huebner, R., Porat-Shliom, N., Weigert, R., Wu, W.W., Shen, R.F., and Donaldson, J.G. (2009). Discovery of new cargo proteins that enter cells through clathrin-independent endocytosis. Traffic *10*, 590–599.

31. Thankamony, S.P., and Knudson, W. (2006). Acylation of CD44 and its association with lipid rafts are required for receptor and hyaluronan endocytosis. J. Biol. Chem. *281*, 34601–34609.

32. Yu, K.S., Lin, M.M., Lee, H.J., Tae, K.S., Kang, B.S., Lee, J.H., Lee, N.S., Jeong, Y.G., Han, S.Y., and Kim, D.K. (2016). Receptor-meditated endocytosis by hyaluronic acid@Superparamagnetic nanovetor for targeting of CD44-overexpressing tumor cells. Nanomaterials (Basel) *6*, 149.

33. Perrot, G., Langlois, B., Devy, J., Jeanne, A., Verzeaux, L., Almagro, S., Sartelet, H., Hachet, C., Schneider, C., Sick, E., et al. (2012). LRP-1–CD44, a new cell surface complex regulating tumor cell adhesion. Mol. Cell Biol. *32*, 3293–3307.

34. Aguiar, D.J., Knudson, W., and Knudson, C.B. (1999). Internalization of the hyaluronan receptor CD44 by chondrocytes. Exp. Cell Res. *252*, 292–302.

35. Chang, G., Zhang, H., Wang, J., Zhang, Y., Xu, H., Wang, C., Zhang, H., Ma, L., Li, Q., and Pang, T. (2013). CD44 targets Wnt/β-catenin pathway to mediate the proliferation of K562 cells. Cancer Cell Int. *13*, 117.

36. Ouhtit, A., Madani, S., Gupta, I., Shanmuganathan, S., Abdraboh, M.E., Al-Riyami, H., Al-Farsi, Y.M., and Raj, M.H. (2013). TGF-β2: a novel target of CD44-promoted breast cancer invasion. J. Cancer *4*, 566–572.

37. Janda, E., Lehmann, K., Killisch, I., Jechlinger, M., Herzig, M., Downward, J., Beug, H., and Grünert, S. (2002). Ras and TGF[beta] cooperatively regulate epithelial cell plasticity and metastasis: dissection of Ras signaling pathways. J. Cell Biol. *156*, 299–313.

38. Suda, K., Murakami, I., Yu, H., Kim, J., Tan, A.C., Mizuuchi, H., Rozeboom, L., Ellison, K., Rivard, C.J., Mitsudomi, T., and Hirsch, F.R. (2018). CD44 facilitates epithelial-to-mesenchymal transition phenotypic change at acquisition of resistance to EGFR kinase inhibitors in lung cancer. Mol. Cancer Ther. *17*, 2257–2265.

39. Bialkowska, A.B., Yang, V.W., and Mallipattu, S.K. (2017). Krüppel-like factors in mammalian stem cells and development. Development *144*, 737–754.

40. Li, Y., Sun, Q., Jiang, M., Li, S., Zhang, J., Xu, Z., Guo, D., Gu, T., Wang, B., Xiao, L., et al. (2019). KLF9 suppresses gastric cancer cell invasion and metastasis through transcriptional inhibition of MMP28. FASEB J. *33*, 7915–7928.

41. Bagati, A., Moparthy, S., Fink, E.E., Bianchi-Smiraglia, A., Yun, D.H., Kolesnikova, M., Udartseva, O.O., Wolff, D.W., Roll, M.V., Lipchick, B.C., et al. (2019). KLF9-dependent ROS regulate melanoma progression in stage-specific manner. Oncogene *38*, 3585–3597.

42. Brown, A.R., Simmen, R.C., Raj, V.R., Van, T.T., MacLeod, S.L., and Simmen, F.A. (2015). Krüppel-like factor 9 (KLF9) prevents colorectal cancer through inhibition of interferon-related signaling. Carcinogenesis *36*, 946–955.

43. Huang, C., Li, J., Zhang, X., Xiong, T., Ye, J., Yu, J., and Gui, Y. (2020). The miR-140-5p/KLF9/KCNQ1 axis promotes the progression of renal cell carcinoma. FASEB J. *34*, 10623–10639.

44. Zhou, Q., Tian, W., Jiang, Z., Huang, T., Ge, C., Liu, T., Zhao, F., Chen, T., Cui, Y., Li, H., et al. (2021). A positive feedback loop of AKR1C3-mediated activation of NF-κB and STAT3 facilitates proliferation and metastasis in hepatocellular carcinoma. Cancer Res. *81*, 1361–1374.

OMTO, Volume *24*

Supplemental information

SNX5 suppresses clear cell renal cell carcinoma progression by inducing CD44 internalization and epithelial-to-mesenchymal transition

Qingqing Zhou, Jiajun Li, Chao Ge, Jinsi Chen, Wei Tian, and Hua Tian

SUPPLEMENTARY MATERIAL

Supplementary Table S1. The sequences of shRNA Target

Identifier	Forward (5’-3’)
SNX5-1	CCCTCATTGACTATGAGAACT
SNX5-2	CCCGTAGTTCGTCTTTAGTTA
KLF9-1	GCTTTGTATGAGTTGTACTTT
KLF9-3	CTCCCATCTCAAAGCCCATTA

SupplementaryTable S2. The sequences of gene-specific primers used for qRT-PCR vector constructs and ChIP assay

Gene name	Forward (5'-3')	Reverse (5'-3')
**Primers for qRT-PCR**		
SNX5	GCACACAAAGACCACACTGC	TGCATCTTCTCTCGAGGACC
CD44	CCATCCCAGACGAAGACAGT	GGTTGTGTTTGCTCCACCTT
Oct4	CTTGCTGCAGAAGTGGGTGGAGGAA	CTGCAGTGTGGGTTTCGGGCA
KLF9	GTGACCACCGAATCTGGGTC	GGCCGTTCACCTGTATGCA
**Primers for vector constructs**		
SNX5(-1297/-30)	GCAAAGCATCTTCCTCCACC	CTCAGCCCGAGTCCAAGATG
**Primers for ChIP**		
ChIP-1	GCCACACAGTCCTAGGTGAC	GCGGTCACAGTAATGGGAC
ChIP-2	GAATGAACCCGTTGCACAGA	AATCCAGACTCCTGCCACAG

Supplementary Table S3. Antibodies used in this study.

Antibody	catalog	Dilution	Company
**For Western blotting**			
SNX5	ab180520	1:500	Abcam
MMP9	Ab38898	1:300	Abcam
E-cadherin	3195T	1:300	CST
N-cadherin	13116T	1:800	CST
Zo-1	8193T	1:500	CST
Claudin-1	13255T	1:500	CST
Snail	3879T	1:500	CST
CD44	Sc-7297	1:300	Santa Cruz
Oct4	CST-2750	1:500	CST
MMP2	10373-2-ap	1:300	Proteintech
MMP7	10374-2-AP	1:300	Proteintech
KLF9	A7196	1:300	Abclonal
β-actin	A3854	1:20000	Sigma
Secondary antibody	HRP conjugated goat anti-rabbit IgG	1:4000	Sigma
Secondary antibody	HRP conjugated goat anti-mouse IgG	1:4000	Sigma
**For Immunohistochemistry**			
SNX5	17918-1-AP	1:50	Proteintech
Ki67	Ab16667	1:50	Abcam
PCNA	Ab29	1:10000	Abcam
CD44	Sc-7297	1:50	Santa Cruz
Secondary antibody	Envision kit (HRP, rabbit/mouse, DAB+)	Ready-to-use	DAKO
**For Immunofluorescence staining**			
E-cadherin	20874-1-AP	1:50	Proteintech
N-cadherin	Ab98952	1:50	Abcam
CD44	Sc-7297	1:30	Santa Cruz
Secondary antibody	Alexa Fluor 488 anti-mouse IgG	1:50	Invitrogen
Secondary antibody	Alexa Fluor 594 anti-rabbit IgG	1:50	Invitrogen
**For flow cytometryanalysis**			
E-cadherin	324107	Ready-to-use	Biolegend
CD44	338805	Ready-to-use	Biolegend

Supplementary Figures

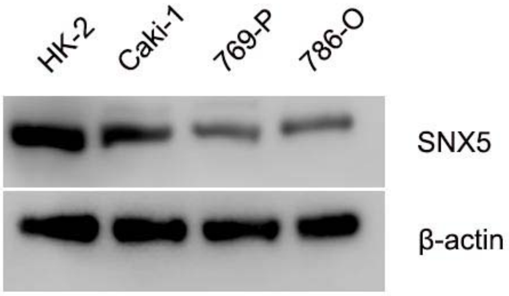

Supplementary Figure S1. The expression of SNX5 was detected by Western blotting in ccRCC cell lines and normal non-malignant renal cell line HK-2.

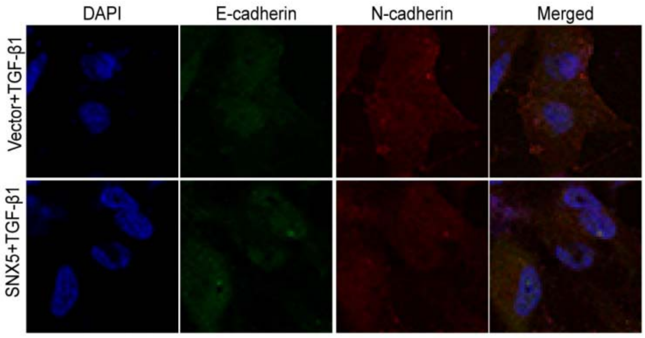

Supplementary Figure S2. SNX5-overexpressing 769-P cells with TGF-β treated were applied to immunofluorescence staining. Antibodies against E-cadherin and N-cadherin were used.

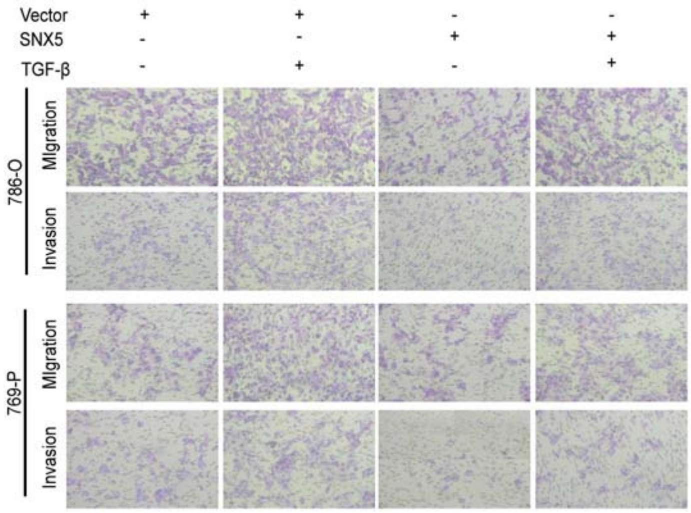

Supplementary Figure S3. SNX5-overexpressed ccRCC cells were treated with TGF-β or control, and cell migration and invasion were evaluated by a transwell assay.

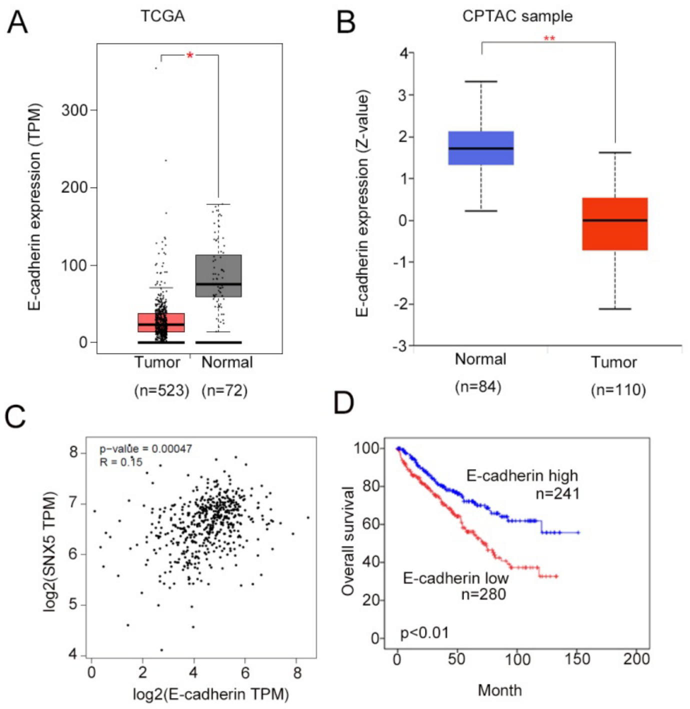

Supplementary Figure S4. **E-cadherin is downregulated and is associated with poor prognosis in ccRCCpatients.**(A) The expression of E-cadherin in ccRCC tissues compared with adjacent normal tissues was analyzed using data sets from TCGA. (B)The expression of E-cadherin in ccRCC tissues compared with adjacent normal tissues was analyzed using data sets from CPTAC. (C) The correlation between SNX5 and E-cadherin in ccRCC tissues using TCGA. (D) Patients with low expression levels of E-cadherin had shorter overall survival than patients with high expression levels as determined using data sets from TCGA.*$p < 0.05$; **$p < 0.01$.

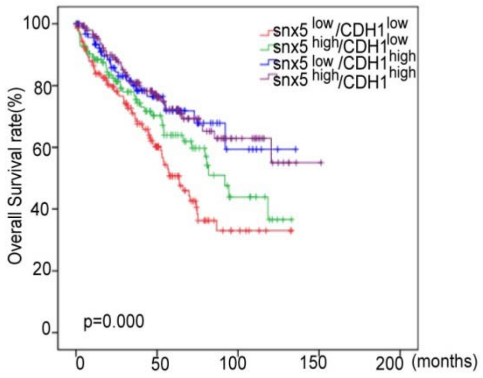

Supplementary Figure S5. Kaplan-Meier analysis of the correlation between the combined expression of SNX5 and E-cadherin with the overall survival of kidney cancer patients according to data sets from TCGA (*P*<0.01, log-rank test).

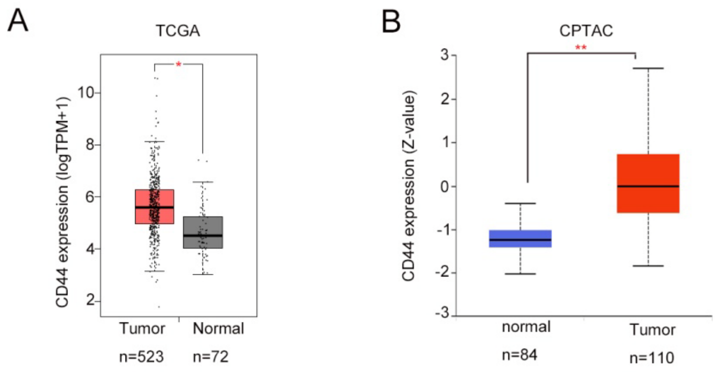

Supplementary Figure S6.(A)The expression of CD44 in ccRCC tissues compared with adjacent normal tissues was analyzed using data sets from TCGA. (B)The expression of CD44 in ccRCC tissues compared with adjacent normal tissues was analyzed using data sets from CPTAC.
